# Green One Health Cardiology: Integrating Nature and Sustainability Into Cardiovascular Prevention and Care

**DOI:** 10.1016/j.jacadv.2025.102368

**Published:** 2025-11-17

**Authors:** Sarayu Chandra Mouli, Coral Lozada, Weichuan Dong, Khurram Nasir, Sarju Ganatra, Karrar Osi, Sanjay Rajagopalan, Jason Fischer, Jay E. Maddock, Sadeer Al-Kindi

**Affiliations:** a Department of Medicine, Center for Health & Nature, Houston Methodist Hospital, Houston, Texas, USA; b Department of Cardiology, Houston Methodist, Houston, Texas, USA; c Sustain Health Solutions, Chelsea, Massachusetts, USA; d Division of Cardiology, Department of Medicine, Lahey Hospital and Medical Center, Burlington, Massachusetts, USA; e Facilities and Planning, MD Anderson Cancer Center, Houston, Texas, USA; f Harrington Heart and Vascular Institute, University Hospitals, Cleveland, Ohio, USA; g Office of Sustainability, Houston Methodist Hospital, Houston, Texas; h School of Public Health, Texas A&M University, College Station, Texas, USA

**Keywords:** cardiovascular health, nature

Cardiovascular disease (CVD) remains the leading cause of morbidity and mortality globally. Improvements in outcomes over past decades have largely resulted from advances in medical and surgical care. However, recent studies suggest these gains have plateaued in the United States.^1^ Comprehensive CVD prevention requires recognition of broader drivers of CVD beyond traditional medical boundaries. Evidence increasingly links CVD outcomes to nature and the environment, affirming the vital role of planetary ecosystems. The Krumholz viewpoint (*JACC Adv*., 2025) caution that further improvement will demand looking beyond traditional clinical boundaries to planetary and environmental drivers.^2^

The One Health framework was developed to conceptualize the interconnectedness of human, animal, and environmental health. Applied to cardiovascular care, it offers a compelling lens for prevention and management. Within this framework, nature is not a passive backdrop but an active determinant of health. Studies show that access to greenspace and walkable environments improves blood pressure, lipids, weight, endothelial function, and psychological well-being, while promoting physical activity and social connection.^3^

Nutrition practices also link ecosystems and cardiovascular health. Plant-forward diets lower risk while conserving the environment while animal-heavy diets drive both heart disease and ecological harm.^4^ At the same time, cardiology itself leaves a footprint. Energy-intensive imaging, invasive procedures, and single-use plastics generate emissions and waste that degrade ecosystems and, ultimately, circle back to CVD risk.^4^ Catheters, tubing, syringes, and personal protective equipment are essential yet nonrecyclable, positioning plastic waste as a novel cardiovascular risk factor.

This viewpoint introduces a Green One Health framework for cardiology, organized into 3 pillars: 1) nature as a health care delivery platform; 2) nutrition as a nature-based intervention; and 3) cardiology’s footprint on nature. These pillars illustrate the bidirectional relationship between heart and planetary health and call on cardiology to embrace environmental stewardship as part of its mission.

## Nature as a health care delivery platform

Nature is an active component of CVD care. Exposure to greenspaces reduces atherosclerosis, improves blood pressure and weight, and supports psychological well-being.^5^ Walkable, biophilic environments also encourage activity and social connection, benefits that extend to neighborhood design, and urban planning. Viewing nature as a health care delivery platform highlights its role as both a preventive and therapeutic tool, and it invites cardiovascular practitioners to integrate nature into clinical strategies, rehabilitation programs, and advocacy for healthier communities.^6^ Nature-positive solutions are replacing “green interventions” by focusing on practical actions rooted in nature, not just carbon reduction.^7^

### Prevention and management through access to greenspace

Access to greenspace is increasingly recognized as a cornerstone of cardiovascular prevention and management. Neighborhoods with higher tree cover, connected green corridors, and accessible parks are consistently associated with lower rates of hypertension, coronary artery disease, and CVD mortality.^3^ These environments encourage physical activity, reduce exposure to pollutants, and provide psychological restoration that lowers stress-related neurohormonal activation.^7^ Incorporating nature-based features into prevention and rehabilitation strategies, such as cardiac rehabilitation programs held in outdoor settings, offers a pragmatic and low-cost approach to improving cardiovascular outcomes.

### Nature prescriptions

Nature contact has recently been conceptualized as a health behavior like physical activity and healthy eating. Growing “nature prescriptions” represent a simple but powerful clinical intervention. These prescriptions are structured recommendations from clinicians to spend time in nature. This enables practices to build evidence-based databases of nature on CVD care.^6^ Shinrin-Yoku (forest-bathing) in Japan exerts powerful medicinal properties through natural compounds released by trees. These prescriptions have already seen success in various practices. Embedding them into cardiac rehabilitation or hypertension management amplifies medical therapy with a simple, nonpharmacologic tool.

### Built environment and urban design

Nature can extend beyond altering individual behaviors to the design of communities. Physical environments that prioritize walkability, connected sidewalks, greenways, and safe public spaces foster physical activity and reduce sedentary time.^6^^,^^7^ Collaboration between health care professionals, urban planners, and policymakers is essential to design environments that promote cardiovascular health at scale. Initiatives that expand green infrastructure, such as park revitalization, not only improve cardiovascular outcomes but also reduce heat exposure, enhance social cohesion, and contribute significantly to climate resilience.^7^ By advocating for healthy design, cardiologists can help shape environments that make cardiovascular prevention a default choice.

### Biophilia in health care architecture

Biophilic design integrates natural elements such as natural light, indoor plants, water elements, and views of greenery into the built environment has been shown to improve various health outcomes including cardiovascular health.^8^ These elements can reduce stress, lower blood pressure, and improve recovery in hospitalized patients.^8^ For health care workers, biophilic design has been shown to mitigate burnout, enhance cognitive performance, and improve job satisfaction, which indirectly benefits patient care.^8^ In cardiology, where stress, anxiety, and prolonged recovery are common, embedding biophilic principles into hospitals and clinics transforms the care environment into a therapeutic ally. By aligning health care architecture with the restorative qualities of nature, biophilic design not only supports cardiovascular outcomes but also embodies the One Health principle that human well-being is inseparable from the health of the environments in which care is delivered.

## Nutrition as a nature-based intervention

Food is one of the most direct connections between people and nature, and dietary choices profoundly shape cardiovascular health. Agricultural practices and food systems link ecosystems with human well-being.^4^^,^^9^ For cardiologists, nutrition offers a powerful lever for both prevention and management of disease. Plant-forward diets not only reduce cardiovascular risk but also protect the environment by conserving land, water, and biodiversity.^4^ Framing nutrition as a nature-based intervention emphasizes that healthy diets benefit both patients and the ecosystems that sustain them.

### Plant-forward diets

Plant-forward diets rich in whole grains, fruits, vegetables, legumes, nuts, and seeds reduce risk of atherosclerosis, hypertension, diabetes, and major cardiovascular events.^10^ These foods provide fiber, antioxidants, and micronutrients that improve vascular health and reduce inflammation. Clinical and epidemiologic studies, including large cohorts and randomized trials, demonstrate that diets such as the Mediterranean or Dietary Approaches to Stop Hypertension diet are associated with lower rates of myocardial infarction, stroke, and cardiovascular mortality. Encouraging patients to adopt such diets complements pharmacology and remains a cornerstone of prevention.^10^

### Sustainability link

The benefits of plant-based nutrition extend beyond individual health to planetary health. Compared with diets high in animal products, plant-forward diets require less land, water, and energy, and generate substantially lower greenhouse gas emissions, contributing to global sustainability goals.^3^ Recommending plant-forward diets, therefore, offers a dual benefit: improving cardiovascular outcomes while contributing to the preservation of ecosystems that support long-term population health. Evidence from large U.S. cohorts, with 30+ years of follow-up, links adherence to planetary diet principles with both lower mortality and reduced environmental impact.^9^

### Equity and access

To be meaningful, nutrition guidance must also account for barriers faced by patients in food deserts and underserved communities. Structural inequities limit access to fresh produce, drive reliance on ultra-processed foods, and perpetuate disparities in cardiovascular outcomes. Cardiologists and health systems have a role in advocating for policies that improve access to affordable, healthy foods through subsidies, community gardens, and partnerships with local food systems. Acknowledging the structural determinants of diet ensures that nutritional recommendations are equitable, actionable, and aligned with the broader goal of reducing health disparities.

## Cardiology’s footprint on nature

The relationship between cardiovascular health and nature is multidimensional. On one hand, access to natural environments supports prevention and management of CVD. Clinical choices shape emissions, waste, and resource use pressures that reverberate through ecosystems and, in turn, human health.

### Emissions and waste

Energy-intensive imaging and invasive procedures not only generate greenhouse gas emissions but also accelerate climate change, which contributes to the degradation of forests, wetlands, and green spaces that buffer air quality and regulate temperature. Pharmaceutical byproducts and contrast agents often enter waste streams that affect soil, waterways, and biodiversity. In this way, routine cardiovascular care directly alters the ecosystems that sustain human health.^5^

Cardiology is one of the most plastic-intensive specialties: catheters, stents, tubing, syringes, drapes, and packaging are designed for single use. Most end up incinerated or landfilled, releasing greenhouse gases and toxins. Microplastics now found in air, water, and human tissue may worsen endothelial dysfunction, accelerate atherosclerosis, and increase arrhythmic risk. Plastic waste is therefore not just an environmental concern but a novel cardiovascular risk factor. The growing “plastemic” mirrors the cardiometabolic epidemic in scale and urgency, demanding cardiology confront its dependence on single-use devices.

### Sustainable practice

Sustainability efforts in cardiology have the potential to protect both patients and nature. Energy-efficient imaging protocols reduce demand on fossil fuel–based energy sources, slowing the warming that harms ecosystems and green landscapes. Minimizing single-use devices and adopting environmentally conscious supply chains lowers burdens on rivers, oceans, and wildlife. Environmentally conscious supply chains that prioritize reusables, bioplastic alternatives, and circular economy models can help mitigate this plastemic, a parallel epidemic to cardiometabolic disease. Telehealth cuts transport-related emissions and preserves habitats. Recent analyses show that remote cardiac monitoring can significantly reduce the carbon footprint of arrhythmia management, underscoring the environmental benefits of digital health solutions in cardiovascular care.^11^ Embedding sustainability into cardiology ensures clinical excellence while safeguarding natural systems.^5^

### Advocacy

Cardiologists are uniquely positioned to advocate for policies that connect cardiovascular health with the protection of nature. Urban tree planting, green hospital infrastructure, and carbon-neutral policies align clinical outcomes with environmental preservation. Framing emissions reductions as cardiovascular prevention shows that protecting nature directly supports heart health. By speaking to the intertwined health of people and ecosystems, cardiologists can champion a clinical culture that embraces stewardship of nature.

### Toward one health cardiology

**Figure 1:**
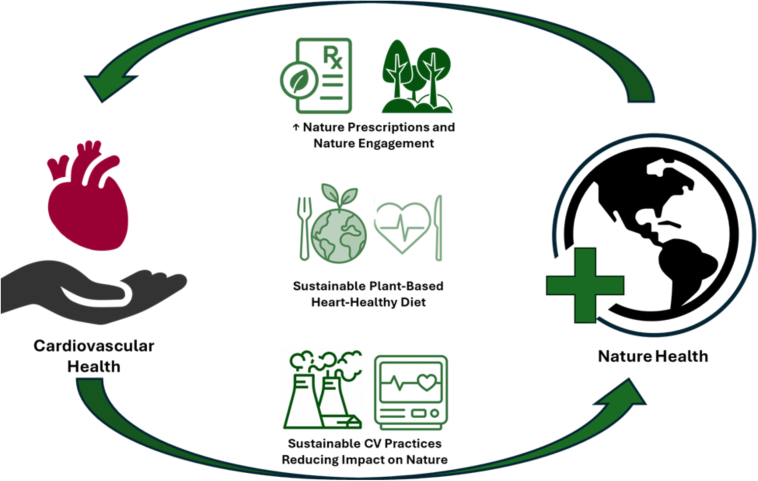
**Integrating Nature and Cardiovascular Health Through a One Health Framework** This conceptual model illustrates a bidirectional relationship between CVD and nature health through a One Health lens. Nature, sustainability, and eco-conscious CVD care form a reinforcing cycle of mutual benefit to integrate environmental stewardship into cardiovascular practice. CV = cardiovascular; CVD = cardiovascular disease.

Integrating nature into cardiovascular practice requires a broader perspective that considers the shared health of individuals, communities, and ecosystems. One Health cardiology reframes heart care as inseparable from the health of communities and ecosystems. Bringing together nature, diet and the environmental impacts is integral to holistic prevention, management, and the future of cardiovascular medicine (Figure 1).

Together, these elements reframe cardiology as not only a field that treats disease but one that supports planetary stewardship. A One Health approach reinforces the idea that protecting ecosystems is essential to protecting heart health. It also aligns cardiovascular medicine with broader goals of sustainability, equity, and resilience. Clinicians can encourage time in nature, prescribe plant-forward diets, and adopt low-impact care practices. Health systems should design environmentally conscious facilities, expand telehealth, and improve access to healthy environments. Professional societies must integrate sustainability into clinical guidelines, recognizing that prevention and environmental protection are deeply connected.

## Funding support and author disclosures

The authors have reported that they have no relationships relevant to the contents of this paper to disclose.
